# Health Disparities: Crisis Not Over for Hurricane Victims

**DOI:** 10.1289/ehp.114-a462

**Published:** 2006-08

**Authors:** Adrian Burton

Thousands of Gulf Coast families displaced in 2005 by Hurricanes Katrina and Rita are the victims of an unprecedented epidemic of chronic medical and mental health problems, yet are receiving little appropriate care, reveals a report released 17 April 2006 from the Mailman School of Public Health. “A year after Katrina, over half of the New Orleans population has not returned—perhaps as many as three hundred thousand people,” says principal investigator David Abramson, acting director of research at the Columbia University National Center for Disaster Preparedness. Many families still live in FEMA-subsidized trailer parks.

Even before the hurricanes hit, Louisiana and Mississippi ranked 50th and 49th in the nation, respectively, in terms of overall health status, according to the United Health Foundation’s *America’s Health: State Health Rankings 2004*. Today, post-hurricane reconstruction has hardly begun. With the loss of hospitals, clinics, pharmacies, medical records, and (for many people) employer-subsidized medical insurance, thousands of residents of what was already one of the nation’s most medically underserved regions could be facing serious long-term health consequences.

Under the auspices of the Louisiana Child & Family Health Study, Abramson’s team used multistage random sampling to select 820 households from 14 FEMA-financed housing sites across Louisiana. More than three-quarters of the households responded, representing 1,171 adults and 488 children. Respondents were interviewed at their homes about chronic medical conditions suffered by their family members, their children’s emotional and behavioral status, their previous and current access to health care services, medical insurance coverage, and the family’s post-hurricane displacement history.

Respondents had moved an average of 3.5 times—some as many as 9 times—with the consequent loss of stability. Thirty-four percent of the children had at least one diagnosed chronic medical condition, a rate one-third higher than the general U.S. child population, with asthma and developmental delays among the most-cited problems. Nearly half the children who had a personal doctor before the hurricanes no longer had one.

Almost 50% of the parents said at least one of their children had emotional or behavioral problems they did not have before they became displaced. And the children aren’t alone: a standardized test given at the time of the interview indicated that more than two-thirds of the mothers interviewed may be suffering depression or anxiety disorders.

Though these families are in acute need of medical surveillance, access to health care resources remains limited. According to a white paper accompanying the report by the Children’s Health Fund, which lobbies for comprehensive health care for all children, only 3 of 9 acute care hospitals that existed in New Orleans before Katrina are now operating at full capacity, and only 19 of 160 clinics remain open. Some 44% of respondents had no medical insurance—about twice as many as before Katrina. In addition, people who were earning above the threshold for receiving Medicaid before Katrina hit were still ineligible for that program since eligibility depends on the previous year’s income.

The white paper calls for Congress and President Bush to establish a “health care Marshall Plan” to address the urgent needs of displaced families. “National leaders need to be aware that this is an unprecedented situation,” says Irwin Redlener, director of the National Center for Disaster Preparedness and president of the Children’s Health Fund. “There needs to be an emergency effort to bring health care professionals to the Gulf region, to rebuild hospitals, to get people’s medical conditions into databases that can be used wherever they end up, to increase mental health benefits under Medicaid, and to bring school-based health services fully online.”

Senator Susan Collins (R–Maine), chairwoman of the Senate committee that investigated the government response to the Katrina disaster, says, “Nearly a year after Hurricane Katrina, Gulf Coast residents are still struggling to return to a sense of normalcy. It is extremely important that the government do all that it can to help address not just this immediate health crisis but all of the long-term needs of those who survived this terrible natural disaster.”

Adds Senator Mike Enzi (R–Wyoming), “We must build on the private and public sector investments in New Orleans and the Gulf Coast, attracting medical personnel as hospitals and health centers are rebuilt, and give survivors the necessary and appropriate assistance to reclaim their lives.”

The authors plan to publish the full text of *On the Edge: Children and Families Displaced by Hurricanes Katrina and Rita Face a Looming Medical and Mental Health Crisis* in a peer-reviewed journal. In the meantime, the report is available free of charge by contacting Abramson at dma3@columbia.edu.

## Figures and Tables

**Figure:**
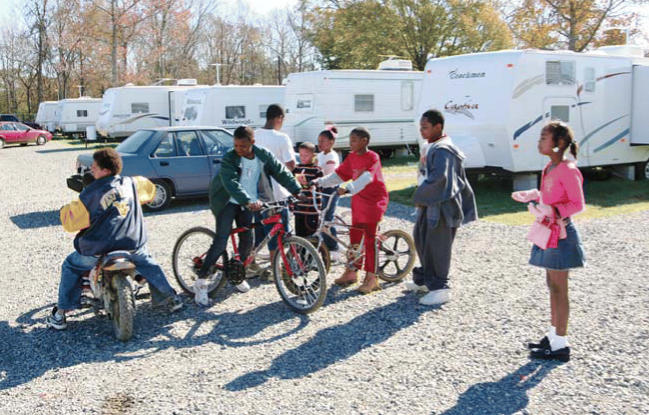
Adding insult to injury Children play in the Baker, Louisiana, FEMA trailer park where they have been living since Hurricane Katrina forced them from their New Orleans homes. A new study shows that many children displaced by the hurricane have lost what small access to health care they had to start with, and therefore are not being treated for a host of medical conditions.

